# Crystal structure of an inferred ancestral bacterial pyruvate decarboxylase

**DOI:** 10.1107/S2053230X18002819

**Published:** 2018-02-26

**Authors:** Lisa Buddrus, Emma S. V. Andrews, David J. Leak, Michael J. Danson, Vickery L. Arcus, Susan J. Crennell

**Affiliations:** a School of Biochemistry, University of Bristol, University Walk, Bristol BS8 1TD, England; b Department of Biology and Biochemistry, University of Bath, Claverton Down, Bath BA2 7AY, England; c School of Science, Faculty of Science and Engineering, University of Waikato, Private Bag 3105, Hamilton 3240, New Zealand

**Keywords:** ancestral sequence reconstruction, pyruvate decarboxylase, lyases, crystal structure, TPP-dependent enzymes

## Abstract

Pyruvate decarboxylase (PDC; EC 4.1.1.1) is a key enzyme in homofermentative metabolism where ethanol is the major product. PDCs are thiamine pyrophos­phate- and Mg^2+^ ion-dependent enzymes that catalyse the non-oxidative decarboxylation of pyruvate to acetaldehyde and carbon dioxide. As this enzyme class is rare in bacteria, current knowledge of bacterial PDCs is extremely limited. One approach to further the understanding of bacterial PDCs is to exploit the diversity provided by evolution. Ancestral sequence reconstruction (ASR) is a method of computational molecular evolution to infer extinct ancestral protein sequences, which can then be synthesized and experimentally characterized. Through ASR a novel PDC was generated, designated ANC27, that shares only 78% amino-acid sequence identity with its closest extant homologue (*Komagataeibacter medellinensis* PDC, GenBank accession No. WP_014105323.1), yet is fully functional. Crystals of this PDC diffracted to 3.5 Å resolution. The data were merged in space group *P*3_2_21, with unit-cell parameters *a* = *b =* 108.33, *c* = 322.65 Å, and contained two dimers (two tetramer halves) in the asymmetric unit. The structure was solved by molecular replacement using PDB entry 2wvg as a model, and the final *R* values were *R*_work_ = 0.246 (0.3671 in the highest resolution bin) and *R*_free_ = 0.319 (0.4482 in the highest resolution bin). Comparison with extant bacterial PDCs supports the previously observed correlation between decreased tetramer interface area (and number of interactions) and decreased thermostability.

## Abstract 2

An ancestral bacterial pyruvate decarboxylase (with an inferred age of 1248 million years) was reconstructed through ancestral sequence reconstruction, synthesized and recombinantly expressed in *E. coli*. The enzyme is fully functional and its crystal structure was elucidated to 3.5 Å resolution.

## 1. Introduction   

Pyruvate decarboxylase (PDC; EC 4.1.1.1) is a thiamine pyrophosphate- and Mg^2+^ ion-dependent enzyme that catalyses the non-oxidative decarboxylation of pyruvate to acetaldehyde and carbon dioxide. Bacterial PDCs are key enzymes in homofermentative metabolism where ethanol is the main fermentation product, and as such are of great interest for biotechnological applications, including second-generation bioethanol production for use as a renewable transport fuel. In recent years there has been growing interest in utilizing bacterial PDCs for high-temperature ethanol-production processes in thermophilic bacteria.

However, our current knowledge of bacterial PDCs is extremely limited, as so far only six bacterial PDCs have been described. These are PDCs from *Acetobacter pasteurianus* (ApPDC; PDB entry 2vbi; D. Gocke, C. L. Berthold, G. Schneider & M. Pohl, unpublished work), *Gluconoacetobacter diazotrophicus* (GdPDC; PDB entry 4cok; van Zyl, Schubert *et al.*, 2014 ▸), *Gluconobacter oxydans* (GoPDC; van Zyl, Taylor *et al.*, 2014 ▸) and *Zymobacter palmae* (ZpPDC; PDB entry 5euj; Buddrus *et al.*, 2016 ▸), the extensively studied *Zymomonas mobilis* PDC (ZmPDC; PDB entries 1zpd, 2wva, 2wvg, 2wvh, 3oei and 4zp1; Dobritzsch *et al.*, 1998 ▸; Meyer *et al.*, 2010 ▸; Pei *et al.*, 2010 ▸; Wechsler *et al.*, 2015 ▸) and a PDC from *Sarcina ventriculi* (SvPDC; Lowe & Zeikus, 1992 ▸), which is the only Gram-positive species known to possess a PDC.

One approach to open up new opportunities for bacterial PDCs adapted to a variety of environments is to exploit the diversity provided by evolution. Ancestral sequence reconstruction (ASR), first envisaged by Pauling & Zuckerkandl (1963 ▸), is a method of computational molecular evolution to infer extinct ancestral protein sequences, which can then be synthesized and experimentally characterized (for a comprehensive review of state-of-the-art ASR methods, see Merkl & Sterner, 2016 ▸). ASR explores a sequence space that has already been screened over evolutionary time spans, thus reducing the nonfunctional space that would otherwise be included in protein libraries generated by random mutagenesis, for example. Thus, ASR has an advantage over random or purely computational approaches in that it limits the ‘design space’ to proteins that are properly folded and have a demonstrable activity (Cole & Gaucher, 2011 ▸; Hobbs *et al.*, 2012 ▸).

Many enzymes reconstructed using ASR show higher thermostability and kinetic activity when compared with modern enzymes (Akanuma *et al.*, 2013 ▸; Groussin *et al.*, 2015 ▸; Hobbs *et al.*, 2012 ▸, 2015 ▸; Perez-Jimenez *et al.*, 2011 ▸; Risso *et al.*, 2013 ▸). Thus, ASR is a promising protein tool for the purpose of generating enzymes and proteins with favourable thermal properties.

In this study, we employed ASR to infer an ancestral bacterial PDC designated ANC27. Here, we present the crystal structure of the inferred ancestral PDC ANC27 at 3.5 Å resolution, show that the enzyme is fully functional and compare it with known extant bacterial PDCs.

## 2. Materials and methods   

### 2.1. Ancestral sequence reconstruction   

#### 2.1.1. PDC sequences   

The ASR input was 25 amino-acid sequences of extant bacterial PDCs retrieved from GenBank or identified through a *BLAST* search based on >50% amino-acid sequence identity over >90% coverage to the reference ZpPDC (PDB entry 5euj). Full details of the strains used and the protein/gene accession numbers can be found in Supplementary Table S1.

#### 2.1.2. Phylogenetic analysis and node-age estimates   

Following the approach used by Hobbs *et al.* (2012 ▸), the PDC amino-acid sequences were aligned using *Geneious* (v.R7 7.1.7; Kearse *et al.*, 2012 ▸) with the *MUSCLE* alignment option (eight iterations) and then refined using *Gblocks* (Castresana, 2000 ▸). *ProtTest* (Abascal *et al.*, 2005 ▸) was used to determine the most appropriate model of amino-acid evolution (WAG + F+). This model and the *Gblocks* alignment were used in *PhyML* (v.3.0; Guindon *et al.*, 2010 ▸) to build a phylogenetic guide tree based on maximum-likelihood (ML) phylogenies. This guide tree, together with the *MUSCLE* alignment, was then implemented in *PRANK* (Löytynoja & Goldman, 2010 ▸) to generate an amino-acid alignment based on phylogenetic information. The resulting alignment was analysed using *GARLI* 2.0 (Zwickl, 2010 ▸) while implementing the WAG model of evolution to find the best ML tree based on log-likelihood scores (see Supplementary Fig. S1). *GARLI* was also used to bootstrap the tree using 1024 replicates. This tree was rooted to the outgroup (PDC sequences with <60% amino-acid identity to ZpPDC, in this case SvPDC with 31% and *Ktedonobacter racemifer* PDC with 52%) in *Geneious*.

The best ML *GARLI* tree was aged using *r*8*s* (v.1.8; Sanderson, 2003 ▸; available at http://loco.biosci.arizona.edu/r8s/). The point of divergence of proteobacteria and firmicutes (3.19 billion years ago) was used as the calibration point, based on a robust prokaryotic phylogeny study using 32 protein sequences and molecular-divergence times estimated from geological calibration points (Battistuzzi *et al.*, 2004 ▸). The resulting tree was visualized in *FigTree* (v.1.4.2; available at http://tree.bio.ed.ac.uk/software/figtree/). See Supplementary Fig. S2 for the maximum-likelihood chronogram.

#### 2.1.3. Ancestral reconstruction   

Three different methods of ancestral inference (amino-acid, nucleotide and codon inference) were performed using the *PAML* software (v.4.3; Yang, 2007 ▸) under the ML criterion. *Pdc* sequences were collated in *Geneious* and manually curated to include gaps as determined by the *PRANK* amino-acid sequence alignment. Nucleotide-sequence inference in *BASEML* used the REV/GTR nucleotide-substitution rate model (with rate variation +I and +G), which was determined to be the most appropriate model of evolution in *JModelTest* (v.2.1.5; Posada, 2008 ▸). GTR was also employed for codon inference in *CODEML*, while for amino-acid inference the WAG model was used. Ancestral gaps were inferred in *PRANK* using the *PRANK* amino-acid alignment and the best ML *GARLI* tree. Taken together, the inferred sequences for nodes of interest were compiled by generating a consensus sequence from all methods of inference. Any ambiguities in the resulting consensus amino-acid sequence were resolved taking the following into consideration: (i) physiochemical properties, (ii) the structural environment, (iii) the most common residues present in extant sequences and (iv) the residue as predicted by the codon-inference method (considered to be the most robust method; Hobbs *et al.*, 2012 ▸).

### 2.2. Macromolecule production   

The amino-acid sequence generated through ASR was backtranslated and synthesized by Eurofins, including XbaI and XhoI restriction sites for cloning into pET-28a(+) (Novagen). The resulting construct added a thrombin cleavage site, a 3× glycine linker and a hexahistidine tag to the C-terminus of ANC27, and was transformed into *Escherichia coli* BL21 (DE3) cells for expression.

For overexpression, the *E. coli* cells were grown in autoinduc­ing Overnight Express TB (Novagen) medium supplemented with 100 µg ml^−1^ kanamycin and 5 m*M* thiamine chloride at 303 K for 16 h with shaking at 220 rev min^−1^.

The cells were harvested by centrifugation (4000*g*, 277 K) and resuspended in His-bind buffer (20 m*M* Tris pH 8, 300 m*M* NaCl, 20 m*M* imidazole). The cells were lysed by sonication on ice and the insoluble debris was removed by centrifugation (17 000*g*, 277 K, 30 min). The supernatant was loaded onto a 5 ml HisTrap HP (GE Healthcare) column for nickel-affinity chromatography. The column was washed with ten column volumes of His-bind buffer before eluting the protein with increasing concentrations of His-elute buffer (20 m*M* Tris pH 8, 300 m*M* NaCl, 1 *M* imidazole) on an ÄKTAexplorer FPLC system (GE Healthcare), monitoring the eluted protein at 280 nm.

The eluted protein was buffer-exchanged into 50 m*M* MES [2-(*N*-morpholino)ethanesulfonic acid] pH 6.5, 20 m*M* MgSO_4_, 3 m*M* TPP using a Superdex 200 10/300 GL gel-filtration column (GE Healthcare) on an ÄKTAexplorer FPLC system and purified to >95% homogeneity as determined by 12% SDS–PAGE analysis.

The enzyme-activity assays were performed as described by Raj *et al.* (2002 ▸), with the reaction mixture consisting of 0.15 m*M* NADH, 20 m*M* MgSO_4_, 3 m*M* thiamine pyrophos­phate (TPP), 29 m*M* pyruvate and 10 U *Saccharomyces cerevisiae* alcohol dehydrogenase (Sigma–Aldrich) in 50 m*M* MES pH 6.5 at 303 K. The reduction in NADH concentration was followed spectrophotometrically at 340 nm. Kinetic properties were analysed using the nonlinear-fit model from the enzyme-kinetics module in *SigmaPlot* (Systat Software). The temperature optimum was determined as described by Gocke *et al.* (2009 ▸) by following the depletion of pyruvate at 320 nm. The denaturation temperature was determined using SYPRO Orange dye in thermal shift assays as described in Huynh & Partch (2015 ▸).

Information relating to the production of ANC27 PDC is summarized in Table 1 ▸.

### 2.3. Crystallization   

Crystals of the purified ANC27 were obtained using the hanging-drop vapour-diffusion method at 291 K. The same approach was used for the crystallization of ANC27 as had been used for earlier PDC structures (Buddrus *et al.*, 2016 ▸). A screen assessing 4 × 96 conditions was carried out in the presence of ligand, and those conditions that resulted in crystal growth were followed up with multiple fine screens to optimize conditions for crystal growth. These efforts did not result in crystals of better diffraction quality. Multiple crystals were screened for diffraction at the home source, and the crystals with the ‘best’ diffraction were further screened at the Australian Synchrotron (AS) for data collection. Following these assessments, further optimization of conditions was carried out; however, the crystal quality did not improve. The data were collected from crystals grown from purified ANC27 that had been incubated with 2 m*M* pyruvate for 30 min at room temperature (293 K). 2 µl drops of the enzyme/pyruvate solution mixed in a 1:1 ratio with crystallization solution [0.15 *M* sodium citrate pH 5.5, 14%(*w*/*v*) PEG 3350] were placed onto cover slips and equilibrated against 400 µl reservoir solution. Crystals were looped out and soaked in cryoprotectant [10%(*w*/*v*) glycerol added to the crystallization buffer] before flash-cooling and storage in liquid nitrogen. Crystallization information is summarized in Table 2 ▸.

### 2.4. Data collection and processing   

X-ray diffraction data were collected to 3.5 Å resolution on the MX2 beamline at the Australian Synchrotron, Melbourne, Australia. *iMosflm* (Battye *et al.*, 2011 ▸), *AIMLESS* (Evans & Murshudov, 2013 ▸) and *BALBES* (Long *et al.*, 2008 ▸) were used for data reduction, data scaling and phasing, respectively. The crystal belonged to space group *P*3_2_21, with unit-cell parameters *a* = *b* = 108.333, *c* = 322.65 Å, and contained two dimers (tetramer halves) in the asymmetric unit. Data-collection and processing statistics are summarized in Table 3 ▸.

### 2.5. Structure solution and refinement   

The structure was solved by molecular replacement in *BALBES* using ZmPDC (PDB entry 2wvg; 71% amino-acid identity) as the starting model. The structure was refined by iterative cycles of manual building and modelling in *Coot* (Emsley *et al.*, 2010 ▸) and refinement in *REFMAC*5 (*CCP*4 suite; Winn *et al.*, 2011 ▸; Potterton *et al.*, 2018 ▸; Murshudov *et al.*, 2011 ▸) and *PHENIX* (Adams *et al.*, 2010 ▸). Refinement in *REFMAC*5 was restricted with noncrystallographic symmetry (NCS) local restraints and TLS (translation, liberation, screw rotation) restraints, and included jelly-body and isotropic temperature factors under automatic weighting. The NCS restraints and TLS parameters were also applied in *PHENIX*. The quality of the final model was checked using *MolProbity* (Chen *et al.*, 2010 ▸). The structure has been submitted to the Protein Data Bank and assigned PDB entry 5npu. Table 4 ▸ summarizes the structure-solution and refinement statistics.

## 3. Results and discussion   

### 3.1. ASR produced a fully functional bacterial PDC   

ANC27 (with an inferred age of 1248 million years; Supplementary Fig. S2) was reconstructed using ASR. The three methods of inference (amino-acid, nucleotide and codon inference) resulted in <3% ambiguous sites, which were resolved as described in §[Sec sec2.1.3]2.1.3. The average posterior probability at each site was 0.6946 (confidence scores for amino-acid, codon and nucleotide inference were 0.89443, 0.43704 and 0.77432, respectively). The final amino-acid sequence showed an amino-acid identity of <79% compared with all extant sequences used in the inference (Supplementary Table S2).

Investigation of the kinetic properties of ANC27 showed a *V*_max_ of 536 ± 13 U mg^−1^ and a *K*_m_ for pyruvate of 3.6 ± 0.3 m*M*. The *K*_m_ is comparable to data from previously characterized extant bacterial PDCs (Buddrus *et al.*, 2016 ▸). However, the *V*_max_ is higher than for any reported bacterial PDC. The highest previously reported *V*_max_ was 181 U mg^−1^, determined for *Z. mobilis* PDC (at pH 6 and 30°C; Bringer-Meyer *et al.*, 1986 ▸). Furthermore, the *k*_cat_ of ANC27 is the highest reported for bacterial PDCs at 580 s^−1^. *k*_cat_/*K*_m_ is very similar to those for GoPDC and GdPDC (Table 5 ▸).

Comparing the thermal properties of ANC27 with those of extant PDCs (summarized in Table 6 ▸), ANC27 is one of the least thermostable and thermoactive bacterial PDCs currently known, only surpassing SvPDC, with which it shares only 33% amino-acid sequence identity.

### 3.2. Crystal structure   

**Figure 1:**
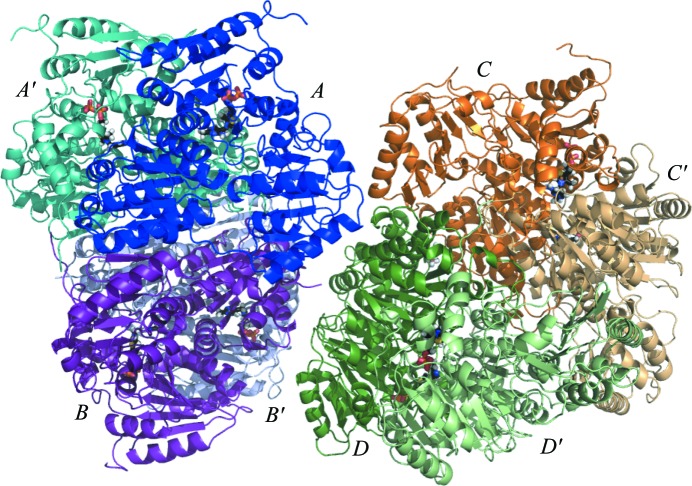
Cartoon representation of ANC27. The four monomers coloured in darker shades of blue (labelled *A*), purple (*B*), green (*D*) and orange (*C*) are present in the asymmetric unit in the final model, while the four outermost monomers coloured pale beige (*C*′), pale green (*D*′), pale violet (*B*′) and pale teal (*A*′) are generated through symmetry. TPP molecules bound between the PYR domain of one monomer and the PP domain of the other are represented as sticks coloured by atom.

**Figure 2:**
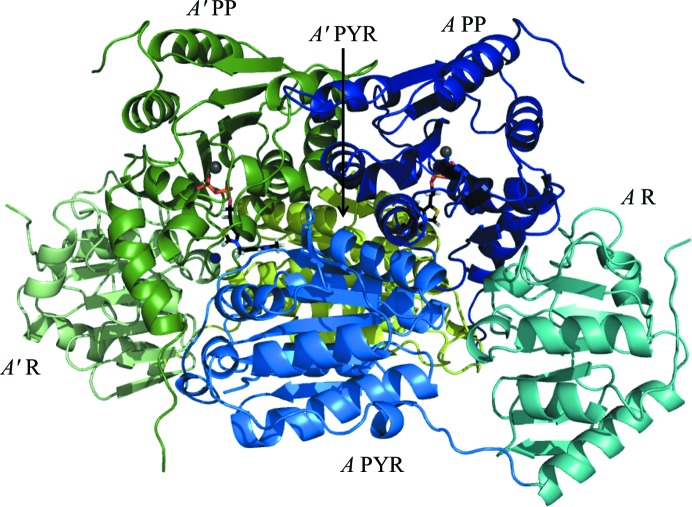
Cartoon representation of an ANC27 dimer. The monomers are coloured by domain. Monomer *A* is coloured blue, with the PYR domain (residues 2–191) in light blue, the R domain (residues 191–361) in teal and the PP domain (residues 362–563) in dark blue. The second monomer (*A*′) is coloured cyan, with the PYR domain in green-cyan and indicated by an arrow, the R domain in pale cyan and the PP domain in dark teal. TPP molecules are represented as sticks coloured by atom. Magnesium ions are represented as grey spheres, while water is shown as a blue sphere.

The crystal structure of ANC27 was determined by molecular replacement using ZmPDC (PDB entry 2wvg; 71% amino-acid identity) as the starting model, with the correct solution confirmed by the presence of electron density for TPP, which was omitted from the search model. As described for extant bacterial PDCs, the quaternary structure of this inferred ancestral PDC is a homotetramer or dimer of dimers (Fig. 1 ▸). The domains can be assigned as follows: amino acids 1–191, PYR (pyrimidine binding, with 177–191 linker); 192–361, R (regulatory, with 341–361 linker); 362–563, PP (pyrophosphate binding) (Fig. 2 ▸). A dimer forms when TPP binds across the two subunits, with the pyrophosphate group binding to the PP domain from one subunit and the pyrimidine ring binding to the PYR domain from the second subunit, thus forming two active sites in the dimer. The final model comprises four chains of 563 amino acids with a molecular mass of 60.6 kDa, each with a TPP and an Mg^2+^ ion, together with one water in the active site of chain *A* and one PEG molecule between chains *A* and *C*. The final model contains four monomers in the asymmetric unit, each comprising part of a dimer formed with a symmetry-related monomer, with the dimers coming together in a less tight interaction to form dimer-of-dimers tetramers. A full tetramer would have a surface area of 65 130 Å^2^.

Superposition of the four monomers yielded an average root-mean-square deviation (r.m.s.d.) of 0.213 Å, indicating that the structures are very similar.

Overall, the electron-density map was of good quality, apart from the N-terminal methionine and the loop 340–360, which is the exposed linker between the R and PP domains. These regions seem to be very flexible and have not been included in the final model (more specifically chain *A* residues 344–358, chain *B* residues 343–359, chain *C* residues 343–357, chain *D* residues 340–358 and the N-terminal methionine in all chains). Compared with other crystal structures of bacterial PDCs ANC27 has much higher average *B* factors (Table 7 ▸). However, in all the PDCs the exposed linker between the R and PP domains is more mobile than the molecule as a whole.

The final model was refined to an *R* factor of 24.6% and an *R*_free_ value of 31.86% using data between 81.1 and 3.5 Å resolution. 92.75% of the residues are located in favoured regions of the Ramachandran plot and a further 6.88% are in allowed regions.

### 3.3. Structural comparison   

It has been noted previously that a trend for increasing tetramer interface areas and increasing numbers of inter­actions at interfaces (*i.e.* forming hydrogen bonds or salt bridges) can be observed as the thermoactivity and thermostability of the PDCs increase (Buddrus *et al.*, 2016 ▸). The interactions between the monomers within a dimer, however, are constant across the thermostability range (45–65°C). This observation is supported by the data obtained from ANC27. Table 8 ▸ summarizes the interface areas and Table 9 ▸ summarizes the numbers of interactions made between different interfaces.

Using *PROMALS*3*D* (Pei *et al.*, 2010 ▸) to generate a structure-based alignment, conserved regions were analysed. Domains are well conserved, but linker regions, especially the linker region between the R and PP domains, are less so. The R–PP linker appears to be extended in ANC27 (Supplementary Fig. S3). However, the diversity displayed by the extant PDCs in the length, sequence and structural positioning of this linker region suggests that it may play a subtle role in the thermostability and thermoactivity differences observed between these PDCs, as previously mentioned by van Zyl, Schubert *et al.* (2014 ▸). GdPDC is one of the least thermostable PDCs, but has one of the shortest linker regions. In ZmPDC this linker is four amino acids longer than the linker in the most thermostable PDC, ZpPDC. The ZpPDC linker is five amino acids shorter than the linker in ANC27.

### 3.4. The active site and TPP binding   

Four monomers form a tetramer with pseudo-222 symmetry and create four active sites. There is good-quality electron-density evidence for a TPP molecule and an Mg^2+^ ion in each of the four chains in the structure. Each TPP binds as indicated by the domain nomenclature, with the pyrophosphate group binding to the PP domain of one monomer and the pyrimidine ring binding to the PYR domain of a second monomer in a dimer, thus creating the active site in a narrow cleft at the interfaces between the two monomers (Fig. 2 ▸).

**Figure 3:**
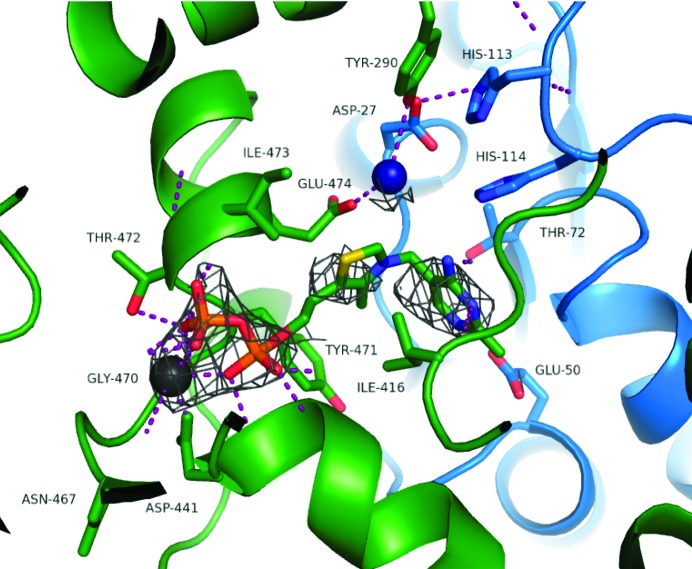
Cartoon and stick depiction of the active site. Residues of one monomer are coloured in green; residues of the other monomer are coloured in blue. The magnesium (dark grey) and water (dark blue) molecules are represented as spheres. The TPP for the green chain is shown as a stick model and coloured by atom. Hydrogen bonds are depicted as magenta dotted lines. The map shown around TPP and magnesium is a simulated-annealing composite OMIT map contoured at 1.5σ.

As noted in PDCs from extant bacteria, TPP is held in a V shape by Ile416 from the first monomer (Fig. 3 ▸ and Supplementary Fig. S4). The O atoms of the pyrophosphate form hydrogen bonds to the backbone amide groups of Ile473 and Thr472, while the N1 atom on the pyrimidine ring forms a hydrogen bond to Glu50 (from the second monomer). These interactions are essential for the catalytic mechanism (Pei *et al.*, 2010 ▸). Tyr471 and Glu474 from one monomer are involved in the binding of the pyrophosphate, while Thr72 from the second monomer is involved in TPP binding around the pyrimidine ring.

Through interactions with Asp27 and Glu474, a water molecule supports the active-site arrangement. This has also been observed in extant PDCs, even in the absence of substrate, and is thought to play a pivotal role in the organization of the substrate complex and of the hydrogen-bond network (Pei *et al.*, 2010).

The Mg^2+^ ion anchors the pyrophosphate group of the TPP and forms interactions with the two O atoms on the diphos­phate group of the TPP, the side-chain O atoms of Asp441 and Asn467 and the main-chain O atom of Gly470. In other PDC structures a water molecule completes the coordination sphere, but in this low-resolution structure this cannot be defined. In all other respects the conformation of TPP and the Mg^2+^ ion are the same in this and the other PDC structures (Supplementary Fig. S4).

### 3.5. Ancestral reconstruction   

This ASR study used an unconventional approach in only using mesophilic species as the inference input to reconstruct a bacterial enzyme, as bacterial PDCs have so far only been found in mesophilic species. Other ASR studies described in the literature use a broader range of species with a wider growth-temperature range, including those by Hobbs *et al.* (2012 ▸) and Groussin *et al.* (2015 ▸).

It is possible that the mesophilic nature of ANC27 is caused by it simply being too young. On close examination of the study conducted by Gaucher *et al.* (2008 ▸) and their description of the progressive cooling of the environmental conditions correlating with a progressive decrease in the denaturing temperature of the bacterial elongation factor TU between 3.5 and 0.5 billion years ago, the denaturing temperature of 62°C for ANC27 fits well with their data. Their data suggest that in ancestors from 1000 to 2000 million years ago, a denaturing temperature of around 50°C is likely to be observed. However, earlier life (3–4 billion years ago) is likely to have experienced conditions similar to today’s hot springs (60–80°C; Gaucher *et al.*, 2008 ▸). There are also reports of fluctuating trends in the thermophily of reconstructed enzymes, in which not all ancestors exhibit the exceptional thermal properties observed for other ancestors (Hobbs *et al.*, 2012 ▸), and evidence for moderately thermophilic ancestors that show no specific trends with regard to catalytic activity or efficiency (Oulavallickal, 2016 ▸). ANC27 maybe more comparable with these enzymes.

In summary, we present the crystal structure of an inferred ancestral bacterial PDC, ANC27, and a brief functional and structural comparison with known extant bacterial PDCs. Structural analysis supports the previously observed correlation between decreased oligomeric interface and salt bridges and decreased thermostability. Although the ancestral protein ANC27 did not possess the thermostability that we hoped for, the suggestion that the lack of inter-dimer interactions appears to be the cause of its fragility should guide future ancestral reconstruction protocols to achieve the desired characteristics.

## Supplementary Material

PDB reference: inferred ancestral pyruvate decarboxylase, 5npu

DNA sequence and Supplementary Figures and Tables.. DOI: 10.1107/S2053230X18002819/or5003sup1.pdf

## Figures and Tables

**Table 1:** Details relating to the production of ANC27 PDC

Source organism	Inferred ancestral sequence
DNA source	Ancestral sequence reconstruction; see Supporting Information for the DNA sequence
Expression vector	pET-28a(+) (Novagen)
Expression host	*E. coli* BL21 (DE3)
Complete amino-acid sequence of the construct produced†	MTYTVGHYLATRLAQIGLKHHFAVAGDYNLVLLDQLLKNKDLEQVYCCNELNCGFSAEGYARANGVGAAVVTFSVGALSAFNAIGGAYAENLPVILISGAPNTNDHGSGHILHHTIGTTDYGYQLEMAKQITCAAVSITHAEDAPALIDHAIRTALREKKPAYIEIACNVAAQPCARPGPVSALLNEPTSDEETLKAAVEAALDFIEKREKPVLLVGGKLRAAGAEEAVVELADALGCAVATMAAAKSFFPEDHPGYVGTYWGEVSSPGVEEIVDWADGIICLGPVFNDYSTVGWTAWPKGENVVLVDPHHITVGGEEFTGIHLKDFLTALTERVPKKDATLDQFKARVGKPAAEKVPAADPNAPLTRAELCRQIQGLLNPNTTLIAETGDSWFNAMRMKLPHGARVELEMQWGHIGWSVPATFGYAVAEPERRNVLMVGDGSFQLTAQEVAQMVRRKLPIIIFLINNRGYTIEVKIHDGPYNNIKNWDYAGLMEVFNAEDGKGLGLKATTGGELAEAIKKALAHREGPTLIECVIDRDDCTPELVTWGKKVATANARPPQAILVPRGSGGGLEHHHHHH

† The C-terminal tag containing a thrombin cleavage site, a 3× Gly linker and a hexahistidine tag is underlined.

**Table 2:** Crystallization conditions

Method	Hanging-drop vapour diffusion
Plate type	24-well
Temperature (K)	291
Protein concentration (mg ml^−1^)	4.5
Buffer composition of protein solution	50 m*M* MES pH 6.5, 20 m*M* MgSO_4_, 3 m*M* TPP, 2 m*M* pyruvate
Composition of reservoir solution	0.15 *M* sodium citrate pH 5.5, 14%(*w*/*v*) PEG 3350
Volume and ratio of drop	2 µl, 1:1
Volume of reservoir (ml)	0.4

**Table 3:** Data collection and processing Values in parentheses are for the outer shell.

Diffraction source	Beamline MX2, AS
Wavelength (Å)	0.9537
Temperature (K)	100.0
Detector	ADSC Quantum 315r CCD
Crystal-to-detector distance (mm)	450
Rotation range per image (°)	1
Total rotation range (°)	101
Exposure time per image (s)	1
Space group	*P*3_2_21
*a*, *b*, *c* (Å)	108.33, 108.33, 322.65
Mosaicity (°)	1.09
Resolution range (Å)	93.82–3.50 (3.71–3.50)
Total No. of reflections	125993 (20708)
No. of unique reflections	28526 (4512)
Completeness (%)	99.5 (99.9)
Multiplicity	4.4 (4.6)
〈*I*/σ(*I*)〉†	5.6 (1.5)
*R*_r.i.m._	0.142 (0.565)
Mn(*I*) half-set correlation CC_1/2_	0.965 (0.422)
Overall *B* factor from Wilson plot (Å^2^)	53.8

† The resolution at which *I*/σ(*I*) falls below 2.0 is 3.79 Å; the cutoff at 3.5 Å was chosen as the ‘maximum resolution’ by *AIMLESS* using CC_1/2_.

**Table 4:** Structure solution and refinement Values in parentheses are for the outer shell.

Resolution range (Å)	81.1–3.5 (3.62–3.50)
Completeness (%)	99.4 (100)
σ Cutoff	*F* > 1.330σ(*F*)
No. of reflections, working set	27079 (2690)
No. of reflections, test set	1358 (122)
Final *R*_cryst_	0.246 (0.3671)
Final *R*_free_	0.319 (0.4482)
No. of non-H atoms	
Protein	16540
Ligand	115 [TPP, 26 atoms each; Mg, 1 atom each; PEG, 7 atoms]
Solvent	1
Total	16656
R.m.s. deviations	
Bonds (Å)	0.007
Angles (°)	0.752
Average *B* factors (Å^2^)	
Protein	87.115
Ligand	85.826
Ramachandran plot	
Most favoured (%)	92.75
Allowed (%)	6.88

**Table 5:** Kinetic properties of known bacterial PDCs in order of increasing thermostability (assay buffer at pH 6.5 unless indicated otherwise)

	SvPDC	ANC27	GdPDC	GoPDC	ZmPDC	ApPDC	ZpPDC
Amino-acid identity (%)	33	Reference	74	66	71	67	69
Kinetics	Sigmoidal	Michaelis–Menten	Michaelis–Menten	Michaelis–Menten	Michaelis–Menten	Michaelis–Menten	Michaelis–Menten
*V*_max_ (U mg^−1^)	45†	536 ± 13	43 (pH 7)‡	125 (pH 7)†	121§	110 ± 2§	165 ± 3¶
							116 ± 2§
*K*_m_ (*S*_0.5_) (m*M*)	5.7†	3.6 ± 0.3	1.2 (pH 7)‡	2.8 (pH 7)††	1.3§	2.8 ± 0.2§	0.67 ± 0.05¶
							2.5 ± 0.2§
*k*_cat_ (s^−1^)	412‡‡	580	NA	125 (pH 7)††	486†	341–508†	341–508†
*k*_cat_/*K*_m_ (*M* ^−1^ s^−1^)	3.2 × 10^4^ ‡‡	1.6 × 10^5^	1.4 × 10^5^ (pH 7.0)‡	1.6 × 10^5^ ††	4.4 × 10^5^ §§	1.3 × 10^6^ (pH 5.0)‡	1.4 × 10^6^ (pH 6.0)‡
PDB code	NA	5npu	4cok	NA	1zpd	2vbi	5euj
GenBank gene ID	AF354297.1	NA	KJ746104.1	KF650839.1	M15393.2	AF368435.1	AF474145.1
GenBank protein ID	AAL18557.1	NA	AIG13066.1	AHB37781.1	AAA27696.2	AAM21208.1	AAM49566.1
R.m.s.d. (Å)	NA	Reference	0.83	NA	0.76	0.87	0.82
*Q* scores	NA	Reference	0.85	NA	0.86	0.84	0.86

† Raj *et al.* (2002 ▸).

‡ van Zyl, Schubert *et al.* (2014 ▸).

§ Gocke *et al.* (2009 ▸).

¶ Buddrus *et al.* (2016 ▸).

†† van Zyl, Taylor *et al.* (2014 ▸).

‡‡ Lowe & Zeikus (1992 ▸).

§§ Siegert *et al.* (2005 ▸).

**Table 6:** Thermal properties of known bacterial PDCs in order of increasing thermostability

	SvPDC†	ANC27	GdPDC†	GoPDC‡	ZmPDC	ApPDC	ZpPDC
Temperature optimum	NA	50°C	45–50°C	53°C	60°C§	65°C§	65°C¶
Temperature dependence of activity retention	45°C: 95%	50°C: 90%	NA (half-life at 60°C 0.3 h)	55°C: 98%	45°C: 85%	50°C: 100%	60°C: 100%
	50°C: 0%	55°C: 38%		60°C: 70%	60°C: 65%	60°C: 65%	65°C: 80%
		60°C: 0%		65°C: 40%	65°C: 45%	65°C: 45%	70°C: 0%¶
					70°C: 0%[Table-fn tfn10]	70°C: 5%[Table-fn tfn10]	
Denaturation temperature	NA	62°C	NA	NA	NA	NA	70°C ††

† Raj *et al.* (2002 ▸).

‡ van Zyl, Schubert *et al.* (2014 ▸).

§ van Zyl, Taylor *et al.* (2014 ▸).

¶ Gocke *et al.* (2009 ▸).

†† Buddrus *et al.* (2016 ▸).

‡‡ Unpublished results.

**Table 7:** Comparison of the flexibility of bacterial PDCs in order of increasing thermostability

	ANC27 (PDB entry 5npu)	GdPDC (PDB entry 4cok)	ZmPDC (PDB entry 1zpd)	ApPDC (PDB entry 2vbi)	ZpPDC (PDB entry 5euj)
Overall *B* factor from Wilson plot (Å^2^)	53.8	14.5	10.4		
Average *B* factors (Å^2^)					
Overall	51.1	15.3	14.9	42.7	26.6
Protein	87.1	13.5	13.2	42.4	26.9
Ligand	85.8	22.6	14.4	38.2	24.6
TPP	85.0	14.2	12.8	38.3	23.5
R–PP linker loop	First and last residue present: 68.4, 70.5	14.9	23.6	43.1	49.6

**Table 8:** Comparison of the interface areas of bacterial PDCs in order of increasing thermostability as calculated using *PDBePISA* (Krissinel & Henrick, 2007 ▸)

	ANC27 (PDB entry 5npu)	GdPDC (PDB entry 4cok)	ZmPDC (PDB entry 1zpd)	ApPDC (PDB entry 2vbi)	ZpPDC (PDB entry 5euj)
Interface area between monomers within a functional dimer (Å^2^)	3947.4	3749.8	4144.5 (4387†)	3761.3	3813.1
Percentage of total monomer surface	18.06	17.83	18.39 (19.4†)	16.95	17.08
Interaction area between two functional dimers to form a tetramer (Å^2^)	1399.4	1851.4	2489.2 (4405†)	2840	2912.16
Tetramer interface as a percentage of total surface of one dimer	6.4	8.79	11.03 (12.1†)	12.78	13.06

† Dobritzsch *et al.* (1998 ▸).

**Table 9:** Comparison of the interactions within interfaces of bacterial PDC structures in order of increasing thermostability; interactions were determined using *PDBePISA* (Krissinel & Henrick, 2007 ▸)

	ANC27 (PDB entry 5npu)		GdPDC (PDB entry 4cok)		ZmPDC (PDB entry 1zpd)		ApPDC (PDB entry 2vbi)		ZpPDC (PDB entry 5euj)	
Interactions on interfaces	Hydrogen bonds	Salt bridges	Hydrogen bonds	Salt bridges	Hydrogen bonds	Salt bridges	Hydrogen bonds	Salt bridges	Hydrogen bonds	Salt bridges
Dimer interface	66	15	63	13	76 (66†)	14 (7†)	61	16	73	12
Major tetramer interface (neighbour‡)	8	0	17	9	29 (64†)	8 (25†)	34	14	31	24
Minor tetramer interface (diagonal‡)	1	0	4	3	6	2	4	0	2	0
TPP pyrimidine ring	10	0	10	0	12	0	10	0	11	0

† Dobritzsch *et al.* (1998 ▸).

‡ Neighbour, interactions between neighbouring monomers; diagonal, interactions between monomers diagonally across tetrameric centre.
