# Supplementary material for: Crystal structure of an inferred ancestral bacterial pyruvate decarboxylase
Source: Acta Crystallogr F Struct Biol Commun. 2018 Feb 26;74(Pt 3):179–86. doi: 10.1107/S2053230X18002819 (PMC5947705; doi:10.1107/S2053230X18002819)
Supplement: Supplementary file 1 [file f-74-00179-sup1.pdf]

STRUCTURAL BIOLOGY COMMUNICATIONS

Volume 74 (2018)

Supporting information for article:

Crystal structure of an inferred ancestral, bacterial pyruvate decarboxylase

Lisa Buddrus, Emma S. V. Andrews, David J. Leak, Michael J. Danson, Vickery L. Arcus and Susan J. Crennell

S1. ANC27 DNA sequence

ATGACGTATACGGTCGGCCATTATTTGGCGACGCGCTTGGCGCAAATTGGCTTGAAACAT
CATTTTGCGGTCGCGGGCGATTATAACTTGGTCTTGTTGGATCAATTGTTGAAAAACAAA
GATTTGGAACAAGTCTATTGCTGCAACGAATTGAACTGCGGCTTTAGCGCGGAAGGCTAT
GCGCGCGCGAACGGCGTCGGCGCGGCGGTCGTCACGTTTAGCGTCGGCGCGTTGAGCGC
GTTTAACGCGATTGGCGGCGCGTATGCGGAAAACTTGCCGGTCATTTTGATTAGCGGCGC
GCCGAACACGAACGATCATGGCAGCGGCCATATTTTGCATCATACGATTGGCACGACGG
ATTATGGCTATCAATTGGAAATGGCGAAACAAATTACGTGCGCGGCGGTCAGCATTACG
CATGCGGAAGATGCGCCGGCGTTGATTGATCATGCGATTCGCACGGCGTTGCGCGAAAA
AAAACCGGCGTATATTGAAATTGCGTGCAACGTCGCGGCGCAACCGTGCGCGCGCCCGG
GCCCGGTCAGCGCGTTGTTGAACGAACCGACGAGCGATGAAGAAACGTTGAAAGCGGCG
GTCGAAGCGGCGTTGGATTTTATTGAAAAACGCGAAAAACCGGTCTTGTTGGTCGGCGG
CAAATTGCGCGCGGCGGGCGCGGAAGAAGCGGTCGTCGAATTGGCGGATGCGTTGGGCT
GCGCGGTCGCGACGATGGCGGCGGCGAAGAGCTTTTTTCCGGAAGATCATCCGGGCTAT
GTCGGCACGTATTGGGGCGAAGTCAGCAGCCCGGGCGTCGAAGAAATTGTCGATTGGGC
GGATGGCATTATTTGCTTGGGCCCGGTCTTTAACGATTATAGCACGGTCGGCTGGACGGC
GTGGCCGAAAGGCGAAAACGTCGTCTTGGTCGATCCGCATCATATTACGGTCGGCGGCG
AAGAATTTACGGGCATTCATTTGAAAGATTTTTTGACGGCGTTGACGGAACGCGTCCCGA
AAAAAGATGCGACGTTGGATCAATTTAAAGCGCGCGTCGGCAAACCGGCGGCGGAAAA
AGTCCCGGCGGCGGACCCGAACGCGCCGTTGACGCGCGCGGAATTGTGCCGCCAAATTC
AAGGCTTGTTGAACCCGAACACGACGTTGATTGCGGAAACGGGCGATAGCTGGTTTAAC
GCGATGCGCATGAAATTGCCGCATGGCGCGCGCGTCGAATTGGAAATGCAATGGGGCCA
TATTGGCTGGAGCGTCCCGGCGACGTTTGGCTATGCGGTCGCGGAACCGGAACGCCGCA
ACGTCTTGATGGTCGGCGATGGCAGCTTTCAATTGACGGCGCAAGAAGTCGCGCAAATG
GTCCGCCGCAAATTGCCGATTATTATTTTTTTGATTAACAACCGCGGCTATACGATTGAA
GTCAAAATTCATGATGGCCCGTATAACAACATTAAAAACTGGGATTATGCGGGCTTGATG
GAAGTCTTTAACGCGGAAGATGGCAAAGGCTTGGGCTTGAAAGCGACGACGGGCGGCGA
ATTGGCGGAAGCGATTAAAAAAGCGTTGGCGCATCGCGAAGGCCCGACGTTGATTGAAT
GCGTCATTGATCGCGATGATTGCACGCCGGAATTGGTCACGTGGGGCAAAAAAGTCGCG
ACGGCGAACGCGCGCCCGCCGCAAGCGATTCTGGTGCCGCGCGGCAGCGGCGGCGGCCT
CGAGCACCACCACCACCACCACTGA

The sequence encoding the additional purification tag is underlined.

Table S1 Sequences used in ASR. *Zymobacter palmae* PDC (PDB entry 5euj) is the reference protein.

Strain	GenBank accession number (amino acid/nucleotide)	% amino acid identity to ZpPDC
Acetobacter aceti	KDE19620.1/JEOA01000009.1	76
Acetobacter malorum	KFL91998.1/JOJU01000008.1	63
Acetobacter nitrogenifigens	WP_026398870.1/NZ_AUBI01000020.1	68
Acteobacter papaya	WP_025859940.1/NZ_BAIN01000005.1	62
Acetobacter pasteurianus	AF368435.1	76
Acetobacter persici	WP_025828656.1/NZ_BAJW01000064.1	69
Acetobacter pomorum	WP_006115789.1/NZ_AEUP01000018.1	76
Acetobacter tropicalis	WP_006559524.1/NZ_BABS01000096.1	62
Acidomonas methanolica	GAJ29946.1/BAND01000085.1	71
Beijerinckia indica	WP_012386294.1/NC_010581.1	65
Commensalibacter sp MX01	EUK18520.1/ATSX01000001.1	56
Gluconacetobacter diazotrophicus	AIG13066.1/KJ746104.1	71
Gluconobacter frateurii	WP_010505240.1/NZ_BADZ01000024.1	70
Gluconobacter morbifer	WP_008852112.1/NZ_AGQV01000006.1	69
Gluconobacter oxydans	KF650839.1	67
Gluconobacter thailandicus	WP_007283613.1/NZ_BAON01000015.1	68
Komagataeibacter europaeus	WP_019085054.1/NZ_CADP01000006.1	65
Komagataeibacter hansenii	WP_003622049.1/NZ_CM000920.1	64
Komagataeibacter medellinensis	WP_014105323.1/NC_016027.1	64
Komagataeibacter oboediens	WP_010515737.1/NZ_CADT01000033.1	66
Kozakia baliensis	WP_029604671.1/NZ_JNAB01000024.1	78
Ktedonobacter racemifer	WP_007922190.1/NZ_ADVG01000005.1	52
Sarcina ventriculi	AF354297.1	31
Zymomonas mobilis	AAA27696.2/M15393.2	63

Table S2 Sequence comparison of ANC27 to all PDCs used as ASR inputs. This information was calculated in Geneious using ClustalW alignments.

	Pairwise amino acid sequence identity in %
Acetobacter aceti	68.4
Acetobacter malorum	74.0
Acetobacter nitrogenifigens	64.7
Acteobacter papaya	73.1
Acetobacter pasteurianus	67.0
Acetobacter persici	66.3
Acetobacter pomorum	68.2
Acetobacter tropicalis	73.1
Acidomonas methanolica	67.5
Beijerinckia indica	77.0
Commensalibacter sp MX01	67.1
Gluconacetobacter diazotrophicus	74.4
Gluconobacter frateurii	67.0
Gluconobacter morbifer	67.8
Gluconobacter oxydans	66.1
Gluconobacter thailandicus	66.7
Komagataeibacter europaeus	76.3
Komagataeibacter hansenii	77.7
Komagataeibacter medellinensis	78.1
Komagataeibacter oboediens	76.7
Kozakia baliensis	70.0
Ktedonobacter racemifer	60.9
Sarcina ventriculi	33.0
Zymobacter palmae	68.8
Zymomonas mobilis	71.1

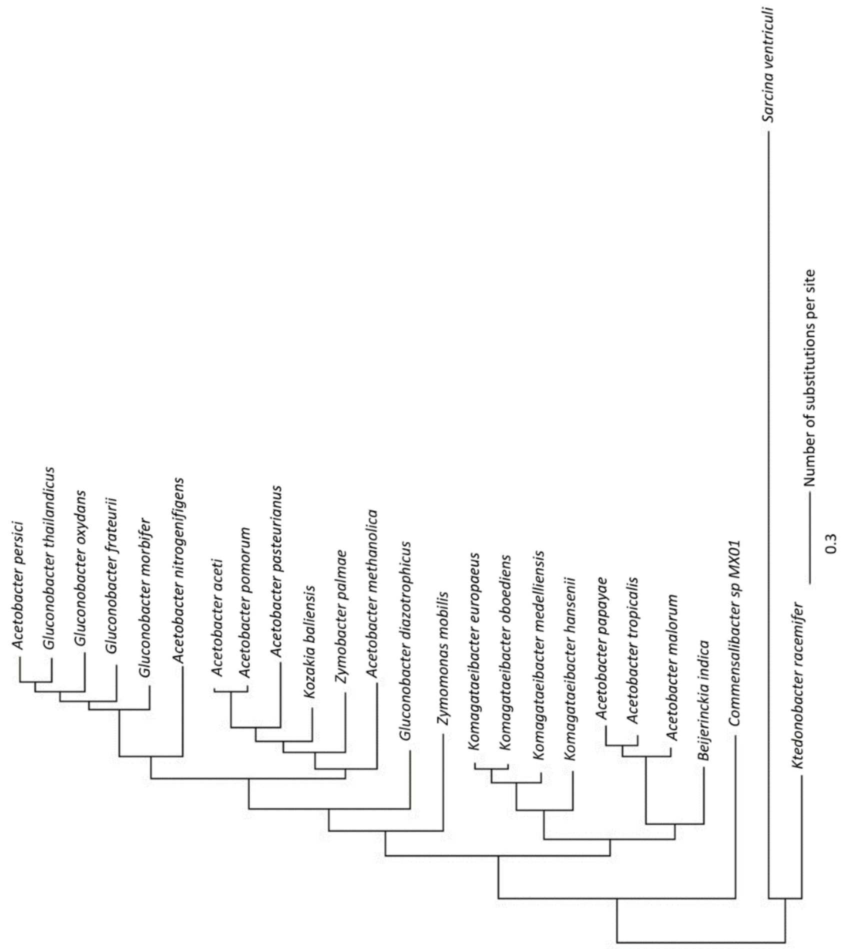

Figure S1 Maximum likelihood phylogram based on PDC amino acid sequences – the best ML GARLI tree. The WAG model of evolution and the PDC amino acid alignment based on phylogenetic information were implemented in GARLI to find the best ML tree. *S. ventriculi* and *K. racemifer* represent the outgroup.

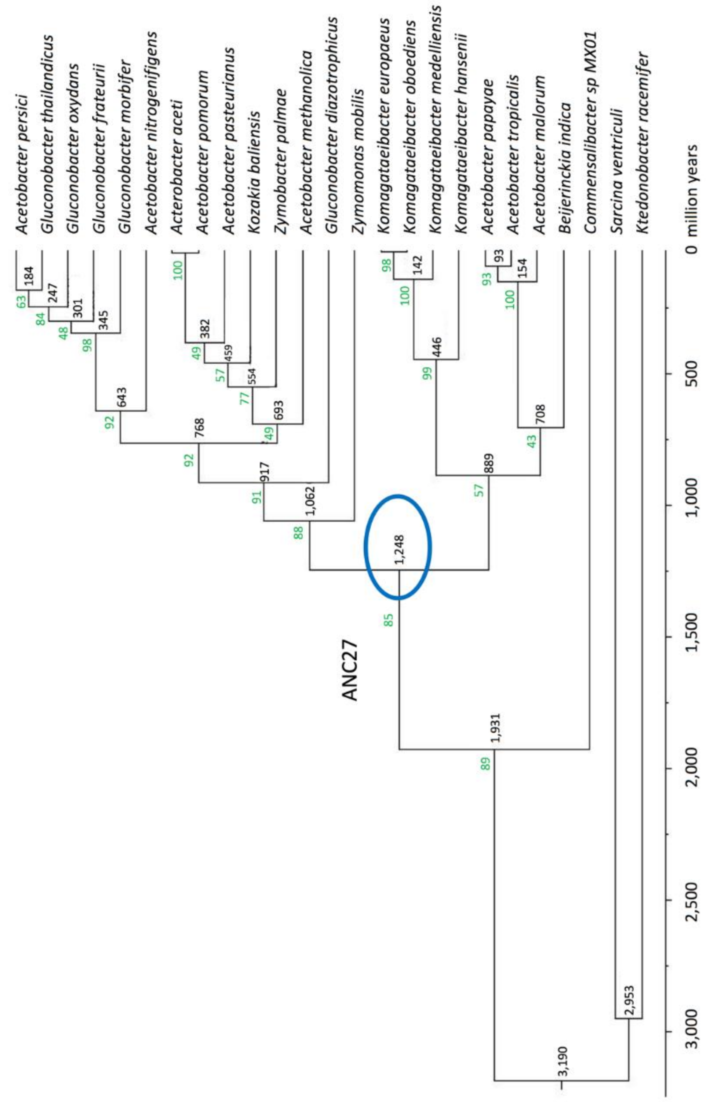

Figure S2 Maximum likelihood chronogram based on PDC amino acid sequences. ANC27 (1,248 million years old) is highlighted in blue. Inferred node ages are indicated in black. Bootstrap percentages were assessed by 1,024 bootstrap replicates and are given in green. *S. ventriculi* and *K. racemifer* represent the outgroup. The point of divergence of Proteobacteria and Firmicutes (3.19 billion years ago) was used as the calibration point for node age estimates.

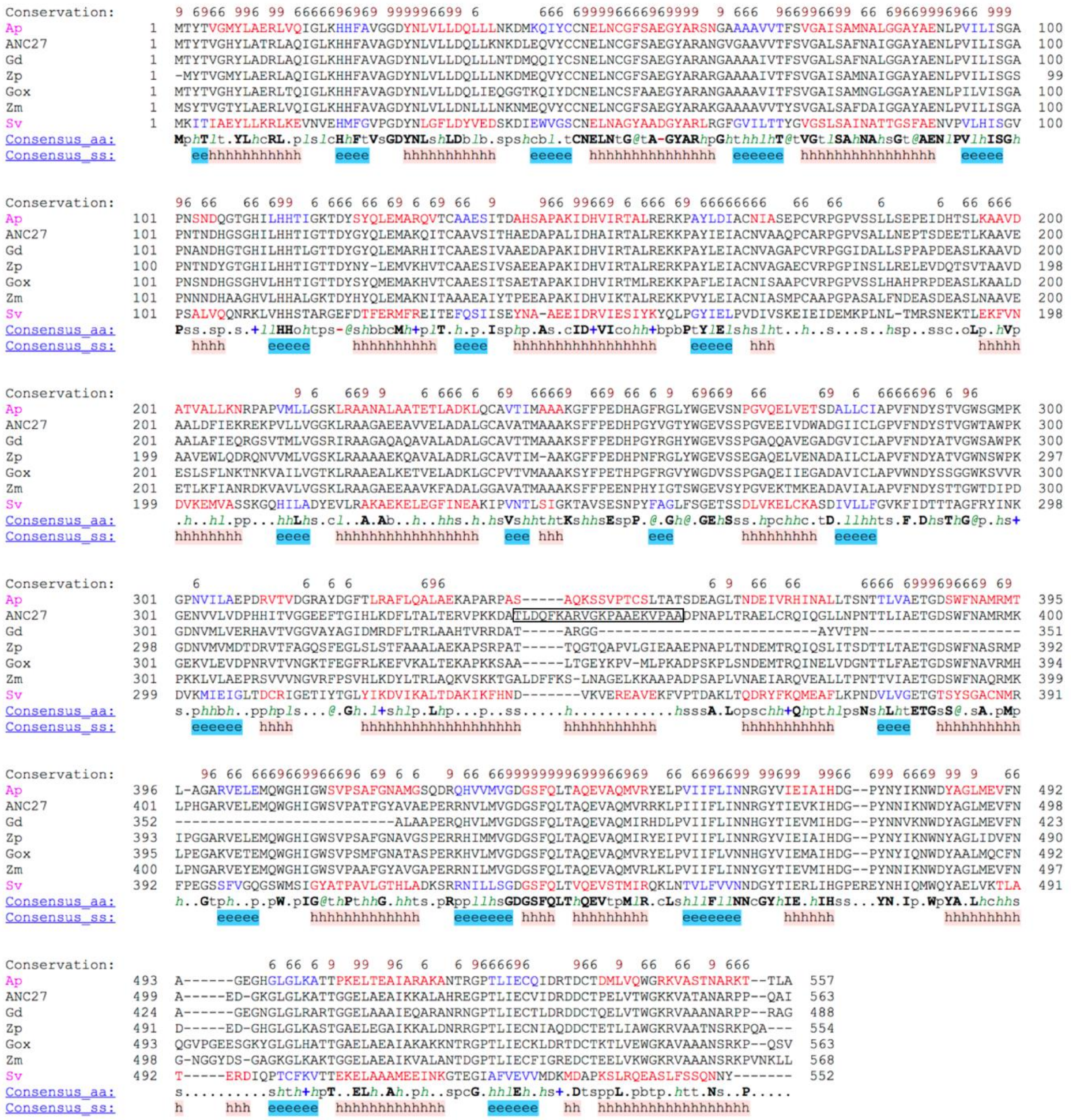

Figure S3 PROMALS3D structural sequence alignment. Each representative sequence has a magenta name and is coloured according to PSIPRED secondary structure predictions (red: alpha-helix, blue: beta-strand). The last two lines show consensus amino acid sequence (Consensus_aa) and consensus predicted secondary structures (Consensus_ss). Consensus predicted secondary structure symbols: alpha-helix: h; beta-strand: e. Consensus amino acid symbols are: conserved amino acids are in **bold** and uppercase letters; aliphatic (I, V, L): *l*; aromatic (Y, H, W, F): @; hydrophobic (W, F, Y, M, L, I, V, A, C, T, H): *h*; alcohol (S, T): o; polar residues (D, E, H, K, N, Q, R, S, T): p; tiny (A, G, C, S): t; small (A, G, C, S, V, N, D, T, P): s; bulky residues (E, F, I, K, L, M, Q, R, W, Y): b; positively charged (K, R, H): +; negatively charged (D, E): -; charged (D, E, K, R, H): c. The ANC27 R-PP linker region is indicated by the black box.

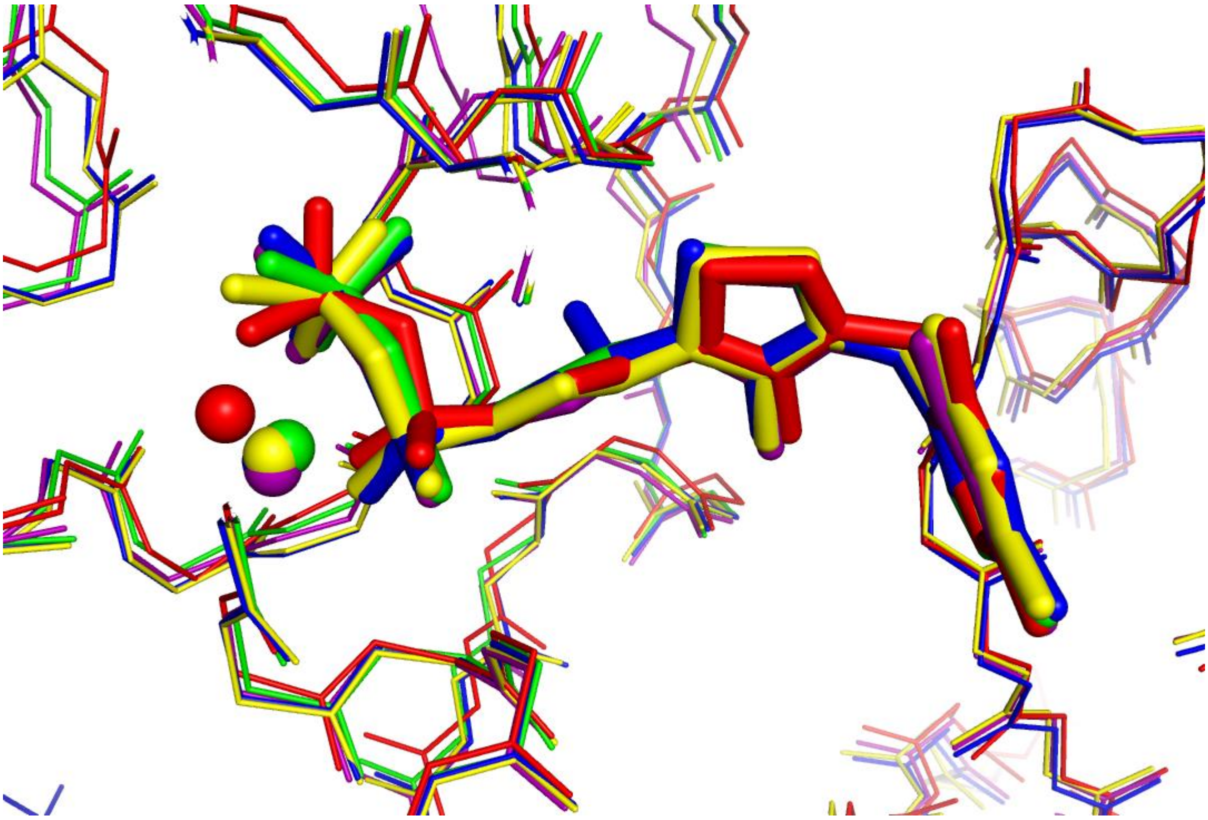

Figure S4 Comparison of ligand geometry. Superposition of bacterial PDCs ANC27 5npu (red), ApPDC 2vbi (purple), GdPDC 4cok (yellow), ZmPDC 1zpd (blue) and ZpPDC 5euj (green). The main chains are displayed as lines, TPP molecules as sticks and magnesium ions as spheres.
